# EFFECT OF HUKOU MOBILITY ON DEPRESSION IN MID- AND LATER LIFE IN URBAN CHINA

**DOI:** 10.1093/geroni/igz038.2244

**Published:** 2019-11-08

**Authors:** Qian Song, James Smith

**Affiliations:** RAND Corporation, Santa Monica, California, United States

## Abstract

We investigate how the hukou system in China creates disparities in psychological well-being among rural-born urban residents in mid- and later-life, using China Health and Retirement Longitudinal Study (2013-2015) and its life history survey (2014). We differentiate merit- (education, employment), policy- (urbanization), and family-based rural-urban hukou converters, and our propensity score analyses show that merit-based (coef.=-0.14,p<0.01) and policy-based (coef.=-0.11,p<0.05) hukou conversion are psychologically protective of the hukou converters over those who still have rural hukou, but family-based conversion does not have an impact. Among all those who realized hukou mobility, those who did so in the period after 1998 has a relative disadvantage. The benefits of policy-based hukou conversion is most prominent for men, childhood converters, and those who realized hukou mobility between 1978 and 1998. Conversion at an older age puts women into disadvantage compared to an earlier age, though this is not the case for men.

